# Genetic Variants Associated with Myocardial Infarction and the Risk Factors in Chinese Population

**DOI:** 10.1371/journal.pone.0086332

**Published:** 2014-01-27

**Authors:** Yongqin Wang, Lefeng Wang, Xin Liu, Yongzhi Zhang, Liping Yu, Fan Zhang, Lisheng Liu, Jun Cai, Xinchun Yang, Xingyu Wang

**Affiliations:** 1 Department of Cardiology, First Affiliated Hospital, Medical College of Shantou University, Shantou, Guangdong, China; 2 Laboratory of Human Genetics, Beijing Hypertension League Institute, Beijing, China; 3 Department of Cardiology, Chaoyang Hospital, Capital Medical University, Beijing, China; 4 School of Basic Courses, Baotou Medical College, Baotou, Neimenggu, China; Yale School of Public Health, United States of America

## Abstract

**Background:**

Recent genome-wide association (GWA) studies in Caucasians identified multiple single nucleotide polymorphisms (SNPs) associated with coronary artery disease (CAD). The associations of those SNPs with myocardial infarction (MI) have not been replicated in Asian populations. Among those previously identified SNPs, we selected nine (rs10953541, rs1122608, rs12190287, rs12413409, rs1412444, rs1746048, rs3798220, rs4977574, rs579459, in or near genes 7q22, LDLR, TCF21, CYP17A1, LIPA, CXCL12, LPA, CDKN2A, ABO, respectively) because of the relatively high minor allele frequencies in Chinese individuals and tested the associations of the SNPs with MI and MI related risk factors in Chinese population.

**Methods and Results:**

We conducted a case–control association study on a cohort of 2365 MI patients and 2678 unrelated controls from the Chinese population. Genotyping of 9 SNPs were performed by the TaqMan Real Time PCR method. After age, sex, and BMI adjustment, we observed the SNPs rs12190287, rs12413409, rs1412444, rs1746048 and rs4977574, were significantly associated with MI in additive models and rs12190287, rs12413409, rs4977574 were significantly associated with phenotypes of MI at the same time. We also found three SNPs rs1122608, rs3798220 and rs579459 were significantly associated with risk factors of MI, although they had no association with MI in Chinese population.

**Conclusion:**

Results of this study indicate that 5 SNPs were associated with MI and 3 SNPs were associated with associated with lipoprotein levels but not with MI in a Chinese population. The present study supports some CAD-related genes in Caucasian as important genes for MI in a Chinese population.

## Introduction

Coronary artery disease (CAD) is one of the leading cause of death and loss of disability-adjusted life years in both developed and developing countries [1]. In China, the World Health Organization estimated that more than 700,000 people die from CAD each year [2]. Myocardial infarction (MI), the most serious clinical manifestation of CAD, is the condition of irreversible necrosis of the heart muscle that results from prolonged ischemia [3]. MI is a complex disease characterized by the inheritance of multiple genetic variants acting in concert with environmental factors to promote the disease state [4]. The INTERHEART study, a standardized global case-control study of risk factors for acute MI had shown that nine easily measured risk factors (dyslipidemia, diabetes, hypertension, abdominal obesity, etc) were associated with more than 90% of the risk of an acute MI worldwide. The population attributable risk (PAR) for these nine risk factors together was 89.9% in Chinese population [5]. In addition, the advent of genome-wide association (GWA) studies led to the identification of several genetic loci that associate with the risk of CAD and MI recently [6]–[9].

Recent GWA studies showed that nine single nucleotide polymorphisms (SNPs) were associated with CAD in Caucasians [10], [11]. These SNPs were rs10953541, rs1122608, rs12190287, rs12413409, rs1412444, rs1746048, rs3798220, rs4977574 and rs579459, which located in or near the 7q22B-cell receptor-associated protein 29(BCAP29) gene, the low density lipoprotein receptor (LDLR)gene, the transcription factor 21 (TCF21)gene, the cyclin M2(CNNM2) gene, the lysosomal acid lipase(LIPA) gene, the chemokine (C-X-C motif) ligand 12 (CXCL12) gene, the lipoprotein, Lp(a)(LPA) gene, the CDKN2A/CDKN2B gene located in 9p21 and the ABO gene, respectively. However, no independent replication studies of these nine SNPs have been reported in the Chinese population with MI. It is very important to identify the associations between genetic variants and risk factors of MI in Chinese population. To discuss the associations of these nine SNPs with MI and risk factors in Chinese population, we genotyped the SNPs in the population based the INTERHEART-China study. We analyzed the associations of the SNPs with MI, as well as with the risk factors for MI in Chinese population.

## Materials and Methods

### Subjects

To assess the importance of risk factors of first acute MI and to clarify whether the effects of risk factors vary in different countries or ethnic groups, a large scale case-control study, the INTERHEART study, was carried out in China and 51 other countries. The present cohort is part of the global INTERHEART study. The Ethics Committee of Beijing Hypertension League Institute approved the study and all participants signed and provided informed consents with the principles outlined in the Declaration of Helsinki. The Ethics Committee approval process was formally documented and with every committee member's signature. The criteria for recruitment of MI cases and controls have been previously described in details [5]. Briefly, cases for INTERHEART study were screened to identify first incident MI, recruited from 24 participating hospitals in 15 cities of China. We included cases without cardiogenic shock or history of major chronic diseases. Acute MI was defined as follows: clinical symptoms and electrocardiogram showing substantial changes such as new pathological Q waves or 1mm ST elevation in any two or more contiguous limb leads, or a new left bundle branch block or new persistent ST-T wave changes diagnostic of a non-Q wave MI, or raised concentration of troponin. Control subjects were recruited from healthy adult visitors to the hospitals without a history of cardiovascular disease, matched for age (up to 5 years older or younger) and sex with the cases. The Ethics Committee of Beijing Hypertension League Institute approved the study and all participants provided informed consents with the principles outlined in the Declaration of Helsinki.

Participants who consented to the study completed a structured questionnaire, attended a health examination, and had a venous blood sample taken. Information about demographic factors, socioeconomic status, lifestyle (smoking, leisure time, physical activity, and dietary patterns), personal and family history of cardiovascular disease, hypertension and diabetes mellitus was obtained. Hypertension and diabetes were defined by self-report of being previously diagnosed or treated with medication for these diseases. Height, weight, waist and hip circumferences, and heart rate were determined by a standard protocol. Blood samples were drawn from every individual, and shipped to a central blood storage site at the Beijing Hypertension League Institute where samples were stored at −160°C in liquid nitrogen.

Plasma concentrations of triglyceride, total cholesterol, high-density lipoprotein cholesterol (HDL-C), apolipoproteins B(ApoB) and A1(ApoA1) were analyzed by Roche/Hitachi 911 analyzer (Roche Diagnostics, Mannheim, Germany), Analytic techniques have been described in detail previously. 5 Total cholesterol, HDL-C and triglycerides were quantified by standard enzymatic procedure (Roche Diagnostics, Mannheim, Germany), and the Assayed Human multi-sera (Randox Laboratories Ltd., UK)was used as quantity control. Immunoturbidimetric assays were used to measure apolipoprotein concentrations (Tina-quant ApoB version 2 and ApoA1 version 2 kits; Roche Diagnostics, Mannheim, Germany). Precinorm and Precipath controls (Roche Diagnostics, Mannheim, Germany) were used in ApoA1 and ApoB analysis in every run. Interassay coefficient variation was less than 5% for all laboratory tests. Low-density lipoprotein cholesterol (LDL-C) concentrations were calculated according to Friedewald's formula [13].

### Selection of SNPs and genotyping

We selected only SNPs in CAD-related genes with minor allele frequencies>0.05 in Chinese in the HapMap database. We used the SNPs effect size in the references, minor allele frequenies of Chinese (based on HapMap database) and sample size of this study to calculate the power for detecting positive association. Exclude the SNPs which the power less than 0.20, we chose nine SNPs (rs10953541, rs1122608, rs12190287, rs12413409, rs1412444, rs1746048, rs3798220, rs4977574, rs579459) that have been shown to significantly associate with CAD but not yet tested in the Chinese population.

Genomic DNAs were extracted from leukocytes using QIAamp DNA Blood Midi Kit (QIAGEN, Germany) according to the manufacture's protocol. SNPs were genotyped by TaqMan Allelic Discrimination Assays with the GeneAmp 7900 Sequence Detection System (Applied Biosystems, Foster City, CA). TaqMan probes were used for genotyping rs10953541 (C_2618842_20), rs1122608 (C_27208850_10), rs12190287 (C_32243431_10), rs12413409 (C_2852843_10), rs1412444 (C_8870364_10), rs1746048 (C_2086883_10), rs3798220 (C_25930271_10), rs4977574 (C_1754681_10), rs579459 (C_26744819_10). Genotyping call rates for all SNPs were greater than 98% (Table S1).

### Statistical analyses

Clinical data about continuous variables expressed as mean±SD, univariate associations were explored with frequency tables and Pearson's χ2 tests for independent proportions. Continuous variables were compared with t-tests or appropriate non-parametric tests, depending on their distribution. Deviation from the Hardy–Weinberg equilibrium in cases and controls were assessed by χ2 analysis [14]. In the case-control analyses allele frequencies in cases and controls were compared using the χ2 test. Odds ratios (ORs) in additive model with corresponding 95% confidence intervals (CI) and adjusted ORs for age, sex and BMI (as independent variables) were performed by logistic regression with allele frequency and genotypes. The associations of SNPs with MI risk factors in the control group were analyzed using the PLINK program [15]. For the MI risk factors analysis, logistic regression and linear regression were carried out, respectively, with age sex and BMI as covariates. The MI risk score was calculated using the weighted sum across the SNPs, combining effect size and doses of risk alleles by the PLINK program. Data were analyzed using SAS statistical software (version 9.2, SAS Institute Inc) and PLINK. P<0.05 was used to indicate statistically significant differences.

## Results

The demographic details for the patients and control subjects are shown in Table 1. Except the means of sex and waist/hip ratio and the frequency of presence of alcohol intake, other characteristics in Table 1 in cases were significantly different from those in controls. To demonstrate the genetic susceptibility of SNPs with MI in the Chinese population, 9 SNPs were genotyped in 2365 MI patients and 2678 controls. All the SNPs were in Hardy –Weinberg equilibrium except rs4977574 which significantly deviated from Hardy –Weinberg equilibrium in control subjects. The genotype information for all these SNPs is available from Supplemental Table 1.

**Table 1:** Characters of the study cohorts.

Characteristic	MI case (n = 2365)	Control (n = 2678)	*P* Value
Age*	60.8 (11.7)	59.5 (11.3)	<0.0001
Sex (Male%)	71.10%	69%	0.162
BMI*	24.7 (3.1)	24.4 (3.0)	0.006
Waist circumference*,cm	86.02 (9.7)	84.8 (9.2)	0.0001
W/H*	0.88 (0.1)	0.88 (0.1)	0.193
History of HT (%)	38.07%	22.59%	<0.0001
History of T2D (%)	11.68%	3.10%	<0.0001
Current smoking (%)	57.67%	39.88%	<0.0001
Alcohol intake (%)	38.67%	36.61%	0.132
HDL-C* mmol/L	1.04 (0.3)	1.10 (0.4)	<0.0001
LDL-C* mmol/L	2.55 (1.3)	2.30 (1.3)	<0.0001
TG* mmol/L	1.62 (1.1)	1.71 (1.1)	0.0115
TC* mmol/L	4.73 (1.2)	4.57 (1.1)	0.010
apoA1* g/L	1.29 (0.3)	1.41 (0.3)	<0.0001
apoB* g/L	0.88 (0.3)	0.83 (0.2)	<0.0001
apoB/apoA1*	0.70 (0.2)	0.61 (0.2)	<0.0001

MI, myocardial infarction; BMI, body mass index (kg/m^2^); cm, centimeters; W/H, waist/hip ratio; HT, hypertension; T2D, type 2 diabetes; HDL-C, high-density lipoprotein cholesterol; LDL-C, low-density lipoprotein cholesterol; TG, triglycerides; TC, total cholesterol; Apo, apolipoprotein.

* Data are means (SD).

Associations between the SNPs and MI are shown in Table 2. There are five (rs12190287 (TCF21) rs12413409 (CNNM2), rs1412444 (LIPA), rs1746048 (CXCL12) and rs4977574 (CDKN2A/CDKN2B)) in nine SNPs were significantly associated with MI in the Chinese population under an additive model. These significant associations remain existed after adjusted for age, sex and BMI. The SNP rs4977574 on 9p21 was most significantly associated with MI in the additive model (OR = 1.50, 95% CI = 1.31 to 1.72, P<0.0001). There were no statistically significant association of rs10953541 (BCAP29), rs1122608 (LDLR), 3798220 (LPA) and rs579459 (ABO) with MI in Chinese population.

**Table 2:** Association between SNPs and MI.

					Additive Model		Additive Model	
				Risk Allele	unadjusted		Adjusted	
rs ID	Gene(s) in region	Minor Allele in Control Subjects	MAF Control: Case		OR(95%CI)	*P* Value	OR(95%CI)	*P* Value
rs10953541	7q22	T	0.17:0.17	C	1.02 (0.90–1.15)	0.746	1.01 (0.90–1.14)	0.846
rs1122608	LDLR	T	0.10:0.09	C	1.02 (0.89–1.18)	0.751	1.02 (0.88–1.18)	0.779
rs12190287	TCF21	G	0.37:0.35	C	1.19 (1.00–1.41)	0.049	1.19 (1.00–1.41)	0.044
rs12413409	CNNM2	A	0.27:0.25	G	1.12 (1.00–1.25)	0.046	1.12 (1.00–1.26)	0.045
rs1412444	LIPA	T	0.32:0.34	T	1.27 (1.06–1.52)	0.010	1.26 (1.05–1.51)	0.013
rs1746048	CXCL12	T	0.37:0.34	C	1.32 (1.10–1.54)	0.002	1.30 (1.09–1.54)	0.002
rs3798220	LPA	C	0.08:0.08	C	1.19 (0.61–2.33)	0.613	1.12 (0.57–2.22)	0.728
rs4977574	CDKN2A/CDKN2B	G	0.44:0.50	G	1.51 (1.32–1.73)	<0.0001	1.50 (1.31–1.72)	<0.0001
rs579459	ABO	C	0.22:0.22	C	1.03 (0.80–1.33)	0.804	1.04 (0.81–1.34)	0.780

**Figure 1:**
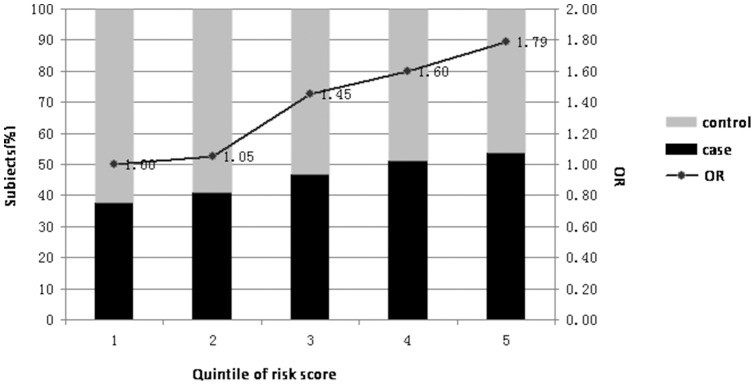
MI risk score categories and risk for MI. Black and gray bars represent the subjects of case and control in each quintile, respectively. Analysis was performed in a total of 4960 individuals (2275 cases, 2675 controls). ORs for MI of the different quantiles compared with those from quintile 1 are shown as solid dots.

To examine the effect of five associated SNPs given in Table 2 (rs12190287, rs12413409, rs1412444, rs1746048 and rs4977574) in aggregate on the risk for MI, a MI risk score was calculated using the weighted sum across the SNPs, combining effect sizes and doses of risk alleles. The mean MI risk score of cases was significantly higher than that of controls (P<2.2×10–16). Logistic regression was applied to test the association of risk score categories with MI. Compared with individuals in the bottom quintile, individuals in the top quintile of MI risk score increased risk for MI (OR = 1.79, 95% CI = 1.48–2.15). The risks for MI across quintiles of MI risk score in shown in Figure 1 and Supplementary Table 2.

We anticipated that some of the genetic risk SNP for CAD would act through established CAD risk factors, including BMI, waist/hip ratio, LDL-C, HDL-C, total cholesterol, triglycerides, ApoA1, ApoB, ApoB/A1 ratio, blood pressure, heart rate, hypertension and type 2 diabetes. Five of nine SNPs were associated with one or more traditional CAD risk factors (Table 3) in our control group. Three (rs579459, rs1122608 and rs3798220) of them have significantly association with the risk factors of CAD, although they have no relationship with MI. The effect allele of rs579459 was associated with increased LDL-C (P<0.020) total cholesterol (P = 0.0008), ApoB (P = 0.003) and ApobB/A1 ratio (P = 0.012). The variants of rs1122608 and rs3798220 were associated with increased triglycerides (P = 0.020, P = 0.040, respectively). The SNP of rs12413409 representing the CYP17A1-CNNM2-NT5C2 gene region was associated with heart rate (P = 0.003) and waist/hip ratio (P = 0.002). The effect allele of rs12190287 was associated with decreased SBP.

**Table 3:** Effects of SNPs on established cardiovascular risk factors.

				Obesity				Lipid														heart rate		blood pressure					Hypertension			Diabetes	
				BMI		W/H		HDL-C mmol/L		LDL-C mmol/L		TC mmol/L		TG mmol/L		apoa1 g/L		apob g/L		apob/apoa1 g/L				SBP mmHg		DBP mmHg							
SNP	Chr	Position	Effect allele	β (SE)	*P*	β (SE)	*P*	β (SE)	*P*	β (SE)	*P*	β (SE)	*P*	β (SE)	*P*	β (SE)	*P*	β (SE)	*P*	β (SE)	*P*	β (SE)	*P*	β (SE)	*P*		β (SE)	*P*		OR(95%CI)	*P*	OR(95%CI)	*P*
rs10953541	7	107031781	C	−0.14 (0.11)	0.187	0.00 (0.00)	0.706	−0.01 (0.01)	0.546	−0.02 (0.05)	0.638	−0.01 (0.05)	0.750	0.02 (0.04)	0.708	−0.01 (0.01)	0.444	−0.01 (0.01)	0.507	0.00 (0.01)	0.954	−0.23 (0.35)	0.524	0.69 (0.64)	0.280		0.54 (0.36)	0.130		1.09 (0.93–1.30)	0.483	1.19 (0.96–1.47)	0.122
rs1122608	19	11024601	C	0.19 (0.14)	0.177	0.01 (0.00)	0.060	−0.03 (0.02)	0.595	−0.16 (0.06)	0.009	−0.04 (0.06)	0.550	0.13 (0.06)	0.020	−0.03 (0.02)	0.086	−0.00 (0.01)	0.812	0.01 (0.01)	0.499	0.75 (0.44)	0.093	−0.06 (0.81)	0.939		0.52 (0.45)	0.244		0.97 (0.80–1.22)	0.856	0.91 (0.70–1.18)	0.471
rs12190287	6	134256218	G	0.07 (0.09)	0.445	0.00 (0.00)	0.830	0.01 (0.01)	0.545	−0.04 (0.04)	0.236	0.01 (0.04)	0.802	0.02 (0.03)	0.629	0.01 (0.01)	0.532	0.00 (0.01)	0.988	−0.00 (0.01)	0.543	0.48 (0.27)	0.078	−1.18 (0.49)	0.017		−0.14 (0.28)	0.612		0.91 (0.86–1.11)	0.273	1.01 (0.86–1.18)	0.942
rs12413409	10	104709086	G	0.06 (0.09)	0.506	0.01 (0.00)	0.002	0.01 (0.01)	0.423	0.05 (0.04)	0.202	0.01 (0.04)	0.894	−0.06 (0.04)	0.141	0.00 (0.01)	0.766	−0.01 (0.01)	0.520	−0.01 (0.01)	0.227	0.87 (0.30)	0.003	0.21 (0.54)	0.700		0.18 (0.30)	0.554		1.03 (0.88–1.15)	0.755	1.00 (0.85–1.20)	0.989
rs1412444	10	90992907	T	0.08 (0.09)	0.382	0.00 (0.00)	0.634	−0.01 (0.01)	0.439	0.05 (0.04)	0.165	−0.00 (0.04)	0.925	0.01 (0.04)	0.725	−0.01 (0.01)	0.146	−0.00 (0.01)	0.863	0.01 (0.01)	0.378	−0.23 (0.28)	0.419	−0.09 (0.51)	0.858		0.24 (0.29)	0.400		0.95 (0.87–1.12)	0.615	1.03 (0.87–1.21)	0.751
rs1746048	10	44095830	T	0.04 (0.08)	0.649	0.00 (0.00)	0.059	−0.01 (0.01)	0.486	0.04 (0.04)	0.278	0.04 (0.03)	0.273	−0.02 (0.03)	0.581	0.00 (0.01)	0.753	0.01 (0.01)	0.095	0.00 (0.01)	0.904	0.07 (0.26)	0.788	−0.40 (0.48)	0.402		−0.05 (0.27)	0.857		0.91 (0.87–1.12)	0.312	0.93 (0.79–1.09)	0.351
rs3798220	6	160881127	T	0.06 (0.15)	0.682	0.00 (0.00)	0.288	0.02 (0.02)	0.260	−0.12 (0.07)	0.060	−0.02 (0.06)	0.761	0.12 (0.06)	0.040	0.01 (0.02)	0.728	0.01 (0.01)	0.558	0.00 (0.01)	0.681	0.06 (0.47)	0.897	0.86 (0.00)	0.401		0.36 (0.48)	0.452		0.86 (0.77–0.19)	0.312	0.82 (0.66–1.19)	0.451
rs4977574	9	22088574	G	−0.03 (0.09)	0.760	−0.00 (0.00)	0.050	−0.01 (0.01)	0.468	0.03 (0.04)	0.450	0.04 (0.04)	0.270	0.04 (0.03)	0.234	−0.01 (0.01)	0.291	0.01 (0.01)	0.166	0.01 (0.01)	0.108	−0.14 (0.27)	0.592	0.03 (0.49)	0.953		−0.06 (0.28)	0.823		0.90 (0.84–1.08)	0.259	1.01 (0.87–1.18)	0.968
rs579459	9	135143989	C	0.06 (0.10)	0.531	0.00 (0.00)	0.550	−0.01 (0.01)	0.374	0.10 (0.04)	0.020	0.14 (0.04)	0.009	0.02 (0.04)	0.632	0.00 (0.01)	0.700	0.02 (0.01)	0.003	0.02 (0.01)	0.012	−0.54 (0.31)	0.085	−0.54 (0.57)	0.345		−0.28 (0.32)	0.373		1.02 (0.93–1.23)	0.844	1.09 (0.96–1.37)	0.651

Chr, chromosome; β(SE), effect size (standard error); OR(95%CI), odds ratio (95% confidence intervals); BMI, body mass index (kg/m2); cm, centimeters; W/H, waist/hip ratio; HT, hypertension; T2D, type 2 diabetes; HDL-C, high-density lipoprotein cholesterol; LDL-C, low-density lipoprotein cholesterol; TG, triglycerides; TC, total cholesterol; Apo, apolipoprotein.SBP, systolic blood pressure; DBP, diastolic blood pressure.

## Discussion

Recent GWA studies have discovered several genetic variants associations with CAD [10], [11]. The present study was carried out in a Chinese population to replicate the positive association signals identified in the GWA studies with CAD in Caucasians. There were significant associations of the SNPs rs12190287, rs12413409, rs1412444, rs1746048 and rs4977574 with MI after age, sex and BMI adjustment in the Chinese population (Table 2).

The SNPs rs12190287, rs12413409, rs4977574 and rs1412444 (located in TCF21, CNNM2, CDKN2A/B and LIPA gene respectively), showed directionally consistent and significantly ORs in both European and Chinese population. The SNPs of rs1412444 and rs4977574 showed stronger associations with MI in our study than CAD patients in Caucasian. This may due to the fact that MI is the most serious clinical manifestation of CAD and showed more significantly association with the related SNPs of CAD.

The SNP rs1746048 in CXCL12 associated with MI in our study, which is same with the result of Schunkert et al research but different from the result of another Chinese population reported by Lu et al [16], which the rs1746048 SNP had no relationship with CAD in Chinese population (OR = 1.01 P = 0.833). This may due to the fact that our study had a large sample size in cases (2365 VS. 1515) and a greater power to detect the association between the SNP of rs1746048 and MI. Another reason may be that our study population was more homogenous than CAD population. All cases in our study had a validated history of myocardial infarction.

In the present study we did not detect statistically significant association of the other four SNPs in the Chinese population. We observed that two SNPs (rs1122608 in LDLR gene and rs3798220 in LPA gene) had low minor allele frequency (MAF≤0.1) in the Chinese population, whereas the MAF of these SNPs were different from the European populations. Because of the low MAFs, a larger sample size will be needed to validate the association of the SNPs and MI in Chinese and other populations. No associations were observed for another two SNPs (rs10953541 in BCAP29 gene and rs579459 in ABO gene), both had MAF greater than 0.1. The observed differences between the results from the Chinese and European populations may partly due to insufficient power or differences in genetic architecture and different gene-lifestyle interactions.

We examined the combined effect of the 5 SNPs on conferring susceptibility to MI which associated with MI in Chinese population, and found that when the person has three of five risk allele, the risk of MI could be increased significantly (Table S2).Our results showed that quintile 5 had a 1.79 times increased odds of MI as compared to quintile 1. Therefore, we suggest that the 5 SNPs are important susceptibility loci for MI in the Chinese population.

In this study we found five of nine SNPs associated with risk factors of MI in Chinese population. The SNPs of rs1122608, rs12190287, rs12413409, rs3798220 and rs579459 were significantly associated with phenotypes of MI (Table 3). Although the SNPs of rs12190287 and rs12413409 have relationships with phenotypes of MI, SBP and heart rate. After adjusting for relevant phenotypes, these SNPs remained significant associations with MI (rs12190287 OR = 1.15, P = 0.044; rs12413409 OR = 1.12, P = 0.045). In the present study we conformed that these two SNPs were independent genetics risk factors of MI and SBP, heart rate were confounding factors by our data. In addition, our results of these two SNPs associated with phenotypes of MI require confirmation in further larger cohorts.

Our study showed that that rs1122608 rs3798220 and rs579459 were associated with LDL-cholesterol levels, triglycerides (TG) levels and plasma cholesterol levels but not with MI in Chinese population. But there was considerable evidence that these three CAD-related SNPs also were associated with MI risk factors in Caucasian. We failed to detect the association may stem from the low allele frequencies (MAF≤0.1 for rs1122608 and rs3798220), the contribution of these SNPs to susceptibility to Chinese MI needs to be evaluated further in larger cohorts. Another explanation is that the participants in the present study were first onset of MI, so that risk factor profile may differ with recurrent CAD patients. The mechanisms of how these loci contribute to susceptibility to MI are still not well understood. Further study is required to elucidate how these loci contribute to conferring susceptibility to MI and risk factors of MI.

The rs1122608 adjacent to LDLR gene has been reproducibly associated with high LDL-cholesterol levels and with an increased risk of myocardial infarction by genome-wide association studies [17]. In present study we verified that rs1122608 was associated with LDL-cholesterol levels and triglycerides (TG) levels. Our data suggest that polymorphisms at LDLR locus may be associated with plasma lipids independently as well as increased risk of MI through raised plasma lipids levels in Chinese population.

The rs3798220 variants in apo(a) gene (LPA) was associated with increase TG level in plasma in our analysis.(Table 3) Several publications also supported that this SNP was associated with both elevated levels of Lp(a) and cardiovascular disease [12], [18], [19]. In present study, we did not have Lp(a) measurement, therefore, we cannot access whether the SNPs is associated with Lp(a).

The ABO SNP rs579459 had strong associations with plasma cholesterol in our data, although this SNP had no relationship with MI. Other GWAS studies also identified ABO as a locus for low-density lipoprotein (LDL-C) [20], type-2 diabetes [21], inflammatory risk biomarkers E-selectin, P-selectin, and sol-ICAM1 [21]–[24]. These findings suggest that ABO might modulate multiple pathways related to cardiovascular risk factors, atherosclerosis and thrombosis.

In our study, we found that many of the replicated genes were associated with MI and risk factors of MI in a Chinese population. Associations of these SNPs likely reflect their roles in development and progression via participation of MI. The functional studies will be required for further tests the hypothesis of their involvement of these genes with MI.

The cases were recruited into the INTERHEART study who suffered their first MI. It is an advantageous to explore the association with lifestyle risk factors since recurrent patients may have altered the lifestyle risk factors after first onset of MI. In addition, all of the patients in our cohort were emergency first MI patients, the cohort would be more homogenous than CAD population. There are limitations of the present study. First, the cohort has limited size, may not have sufficient power to detect SNPs with small effect but true association. The function of selected SNPs were not involved in the study, therefore, cannot provide further details of biological relevance of myocardial infarction. Further studies in other ethnic groups and functional studies are needed. Nevertheless, our results together with results from other population can be used to ascertain the association.

In conclusion, results of the current study indicate that 5 SNPs are associated with MI and 3 SNPs were associated with lipid levels but not with MI in a Chinese population. The present study supports some CAD-related genes in Caucasian as important genes for MI in a Chinese population.

## Supporting Information

Table S1 **The genotyping information of the overall samples.** H-W-*P*, *P* value of Hardy –Weinberg; MAF, minor allele frequency;
(DOCX)

Table S2 **Supplementary Information of** **Figure 1** **.** Quantile,quantile of risk score;Per.case,percent of case number;Per.control,percent of control number;OR, odds ratio; CI, confidence interval;
(DOCX)
