# Supplementary material for: Genetic Variants Associated with Myocardial Infarction and the Risk Factors in Chinese Population
Source: PLoS One. 2014 Jan 27;9(1):e86332. doi: 10.1371/journal.pone.0086332 (PMC3903528; doi:10.1371/journal.pone.0086332)
Supplement: Table S1 — The genotyping information of the overall samples. H-W-P, P value of Hardy –Weinberg; MAF, minor allele frequency; (DOCX) [file pone.0086332.s001.docx]

| Supplemental Table1.The genotyping information of the overall samples | | | | | |  |  |  |  |  |  |  |  |  |  |
| --- | --- | --- | --- | --- | --- | --- | --- | --- | --- | --- | --- | --- | --- | --- | --- |
|  |  |  |  | Case | | |  |  | Control | | |  |  | MAF | |
| rs ID | Gene(s) in region | Minor Allele in Control Subjects | Call Rate | 1 | 2 | 3 | H-W-*P* |  | 1 | 2 | 3 | H-W-*P* |  | Case | Control |
| rs10953541 | 7q22 | T | 97.34% | 1628 | 629 | 73 | 0.201 |  | 1791 | 718 | 70 | 0.847 |  | 0.166 | 0.166 |
| rs1122608 | LDLR | T | 97.90% | 1880 | 404 | 11 | 0.03 |  | 2155 | 465 | 22 | 0.574 |  | 0.092 | 0.096 |
| rs12190287 | TCF21 | G | 98.20% | 981 | 1081 | 271 | 0.304 |  | 1058 | 1208 | 353 | 0.781 |  | 0.348 | 0.365 |
| rs12413409 | CYP17A1,CNNMS,NT5C2 | A | 99.25% | 150 | 870 | 1328 | 0.639 |  | 182 | 1047 | 1428 | 0.596 |  | 0.249 | 0.266 |
| rs1412444 | LIPA | T | 98.14% | 1007 | 1020 | 274 | 0.523 |  | 1234 | 1159 | 255 | 0.472 |  | 0.341 | 0.315 |
| rs1746048 | CXCL12 | T | 98.02% | 990 | 1047 | 262 | 0.554 |  | 1096 | 1168 | 380 | 0.017 |  | 0.342 | 0.365 |
| rs3798220 | LPA | C | 99.21% | 15 | 345 | 1999 | 0.978 |  | 20 | 392 | 2232 | 0.542 |  | 0.079 | 0.082 |
| rs4977574 | CDKN2A,CDKN2B | G | 97.18% | 583 | 1139 | 595 | 0.419 |  | 777 | 1325 | 482 | 0.047 |  | 0.503 | 0.443 |
| rs579459 | ABO | C | 98.39% | 121 | 777 | 1419 | 0.278 |  | 134 | 886 | 1625 | 0.354 |  | 0.22 | 0.218 |
| H-W-*P*, *P* value of Hardy –Weinberg; MAF, minor allele frequency; | | | | | |  |  |  |  |  |  |  |  |  |  |
