# Supplementary material for: Genetic Variants Associated with Myocardial Infarction and the Risk Factors in Chinese Population
Source: PLoS One. 2014 Jan 27;9(1):e86332. doi: 10.1371/journal.pone.0086332 (PMC3903528; doi:10.1371/journal.pone.0086332)
Supplement: Table S2 — Supplementary Information of Figure 1 . Quantile,quantile of risk score;Per.case,percent of case number;Per.control,percent of control number;OR, odds ratio; CI, confidence interval; (DOCX) [file pone.0086332.s002.docx]

| Supplementary Table 2 Supplementary Information of Figue 1 | | | | | |
| --- | --- | --- | --- | --- | --- |
| Quantile | Per.case(%) | Per.control(%) | OR(95%CI) | *P* Value |  |
| 1 | 37.69 | 62.31 | reference |  |  |
| 2 | 40.75 | 59.25 | 1.05(0.87-1.27) | 0.602 |  |
| 3 | 46.81 | 53.19 | 1.45(1.16-1.68) | <0.001 |  |
| 4 | 50.96 | 49.04 | 1.60(1.33-1.93) | <0.001 |  |
| 5 | 53.65 | 46.35 | 1.79(1.48-2.15) | <0.001 |  |
| Quantile,quantile of risk score;Per.case,percent of case number;Per.control,percent of control number;OR, odds ratio; CI, confidence interval; | | | | |  |
|  |  |  |  |  |  |
|  |  |  |  |  |  |
